# Impact of Baseline and Trajectory of Triglyceride-Glucose Index on Cardiovascular Outcomes in Patients With Type 2 Diabetes Mellitus

**DOI:** 10.3389/fendo.2022.858209

**Published:** 2022-03-24

**Authors:** Shi Tai, Liyao Fu, Ningjie Zhang, Ying Zhou, Zhenhua Xing, Yongjun Wang

**Affiliations:** ^1^ Department of Cardiovascular Medicine, The Second Xiangya Hospital of Central South University, Changsha, China; ^2^ Department of Blood Transfusion, The Second Xiangya Hospital of Central South University, Changsha, China

**Keywords:** triglyceride-glucose index, cardiovascular disease, type 2 diabetes mellitus, risk factors, insulin resistance glycated hemoglobin

## Abstract

**Background and Aims:**

This study aimed to evaluate the association of the triglyceride-glucose (TyG) index with the cardiovascular incidence in patients with type 2 diabetes mellitus (T2DM).

**Methods and Results:**

Secondary analysis in patients with long-lasting T2DM from the Action to Control Cardiovascular Risk in Diabetes study was performed. The primary outcome was the first occurrence of major adverse cardiovascular events (MACEs). The association between the baseline and trajectories of the TyG index and MACEs was evaluated by Cox proportional hazards regression analysis. During a median follow-up period of 8.8 years, 1,815 (17.8%) patients developed MACEs. After traditional cardiovascular risk factor adjustments, each 1-standard deviation increase in the TyG index was associated with a 19.00% higher MACE risk, similar to that in the TyG index quartile characterization. Four distinct trajectories of TyG indexes were identified: low (16.17%), moderate (40.01%), high (34.60%), and very high (9.30%). In multivariate analysis, high and very high TyG index trajectories showed a greater risk of future MACE incidence than the low TyG index trajectory. A similar association was observed between the TyG index and the occurrence of coronary heart disease.

**Conclusions:**

The baseline and trajectories of the TyG index were significantly associated with the occurrence of MACEs in patients with T2DM.

**Clinical Trial Registration:**

http://www.clinicaltrials.gov. Unique identifier: NCT00000620.

## Introduction

Type 2 diabetes mellitus (T2DM) is associated with the early onset of atherosclerotic cardiovascular disease (CVD), often presenting as coronary heart disease (CHD), cerebrovascular disease, and peripheral arterial disease in these patients (1). Specifically, patients with diabetes typically develop cardiovascular (CV) abnormalities with greater severity and 14.6 years in advance as compared to individuals without diabetes mellitus (2, 3). Although the incidence of T2DM complications has reduced over the years due to advances in medicine, more than 382 million people worldwide currently have diabetes, making them more vulnerable to CVD-related disability and deaths (4). Therefore, there is a need to prevent CVD in individuals with diabetes. To achieve this goal, it is necessary to develop effective methods to predict and diagnose T2DM-related CVD more accurately in the early stages.

The triglyceride-glucose (TyG) index is related to morbidity and mortality of CVD in the general population (5); it predicts the progression of coronary artery calcification (6). Several studies revealed TyG has associated with insulin resistance (IR) related diseases, such as nonalcoholic fatty liver disease (7) and colorectal cancer (8), as well as implicated in the development of hypertension (9) and atherosclerosis (10). More importantly, previous studies that evaluate associations of the TyG index with a predictor of CVD and adverse CV outcomes are inherently limited by small sample sizes, retrospective study design, or cross-sectional analysis (5, 11–16). Furthermore, only the baseline TyG or TyG index at a single time point was used to study the associations.

To the best of our knowledge, few long-term prospective studies (17, 18) have explored the relationship between adverse CV outcomes and the TyG index in patients with T2DM and its trajectory derived from multiple measurements over time. Accordingly, we hypothesized that dynamic changes in TyG during follow-up may modify the development of adverse CV outcomes. Using the data from the Action to Control Cardiovascular Risk in Diabetes (ACCORD) study (19) and ACCORD Follow-On Study (ACCORDION) (20), we aimed to evaluate the association of the TyG index with adverse CV outcomes and determine the influence of baseline TyG index and different trajectory changes over 10 years on the development of adverse CV outcomes.

## Materials and Methods

### Study Design and Subjects

In this study, a secondary analysis of 10,251 T2DM patients using published data of the ACCORD/ACCORDION trial (ClinicalTrials.gov number, NCT00000620), was performed (21). Briefly, the ACCORD was a 2×2 factorial trial aimed to test whether strict control of blood glucose, blood pressure, and lipids could reduce CVD incidence in T2DM patients. The rationale and design of the ACCORD trial have been described previously. All subjects were recruited from 77 clinical sites across North America between January 2001 and October 2005. The study follow-up was conducted in the ACCORD trial, and closeout visits were completed by June 2009. At the final trial visits, the study participants were invited to participate in the post-trial, non-treatment, observation-only ACCORDION study. The study follow-up ended 60 months after ACCORD (October 31, 2014) for post-trial observation. This ACCORD/ACCORDION study design was approved by the Wake Forest University, USA (coordinating center) and the institutional review boards at each center (participating clinical sites). Written informed consent was obtained from all the participants.

### Exclusion *Criteria*

Participants with missing data for TyG index values at baseline (n=55) were excluded from our study. This resulted in a final sample size consisting of 10,196 participants; they were included for the analysis of the association between the baseline TyG index and primary and secondary outcomes. Participants with fewer than three valid TyG indices during the follow-up visits were also excluded. The remaining 9,697 participants were included for the analysis of the association between the TyG index group-based trajectory and adverse CV outcomes (**Figure S1**).

### Outcomes

The primary outcome of the study was occurrence of major adverse cardiovascular events (MACEs), including non-fatal myocardial infarction (MI), non-fatal stroke, and death from CV causes. The secondary outcomes were all-cause mortality, total stroke, fatal or hospitalized congestive heart failure (CHF)(fatal congestive heart failure was defined as death due to clinical, radiological or postmortem evidence of CHF without clinical or postmortem evidence of an acute ischemic event; hospitalized congestive heart failure was defined as participant with documented clinical and radiological evidence), and major coronary events (CV death, non-fatal MI, or unstable angina). Participants were followed up every 2-4 months *via* telephonic interviews or outpatient clinic visits. Relevant medical information was collected during each follow-up period. The first occurrence of adverse CV events in each patient was determined by the Working Group of the Morbidity and Mortality subcommittee. In addition, the major adverse CV events were also collected with follow-up ended on October 31, 2014, or 60 months after ACCORD, in all patients for a total of 5 years of post-trial observation.

### Data Collection

The data included patient demographic and clinical characteristics, age, sex, ethnicity, education, smoking history, medical history, previous medications, body measurements, and blood content (i.e., fasting glucose, fasting plasma triglycerides (TG), cholesterol, low-density lipoprotein cholesterol [LDL-C], and high-density lipoprotein cholesterol [HDL-C]). Fasting glucose and fasting triglycerides were collected during follow-up visits. The TyG index was calculated as ln (fasting TG [mg/dL] × fasting glucose [mg/dL]/2) (22).

### Statistical Analysis

Normally distributed continuous data are expressed as mean ± SD, non-normally distributed continuous data are expressed as median (interquartile range), and categorical data are expressed as numbers (percentage). Differences among groups were evaluated using analysis of variance or Kruskal–Wallis h-test when appropriate for continuous variables and the χ^2^ test for categorical variables. Kaplan–Meier estimates were used to compute the cumulative incidence of incident MACEs by TyG index quartiles. The differences in the estimates were compared using the log-rank test. A Cox proportional hazards regression model was used to calculate hazard ratios (HRs) and 95% confidence intervals (CIs) between the TyG index and time for MACEs. Three multivariate models with progressive degrees of adjustment were used to adjust for potential confounders of MACEs and adverse CV events. These models were: Model 1, adjusted for baseline age, sex, previous CV event, race, body mass index (BMI), education, systolic blood pressure, and diastolic blood pressure; Model 2: adjusted for model 1 covariates plus baseline estimated glomerular filtration rate (eGFR), hemoglobin A1c (HbA1c), total plasma cholesterol, plasma LDL-C, live alone, duration of diabetes and depression; and Model 3: adjusted for model 2 covariates plus statins, biguanide, aspirin, angiotensin converting enzyme inhibitor/angiotensin-receptor blocker (ACEI/ARB), and insulin.

Furthermore, we used a restricted cubic spline regression model with three knots to assess the nonlinear dose-response association between the baseline TyG index and MACEs. We used latent class models to identify different longitudinal TyG index level patterns within the study participants and tested models with groups ranging from 2 to 5. We examined different criteria, including Bayesian information criteria, to assess the optimal number of trajectories. No group included less than 5% of the participants (see **Table S1**). All the final models classified participants into trajectory groups with good discrimination; the mean probability of final group membership was 0.89. The trajectory group was included as an independent variable in a Cox proportional hazards regression model examining predictors of MACEs at follow-up to estimate the association of TyG index trajectory groups with MACEs. All analyses were conducted using SPSS version 23 (SPSS, Inc., Chicago, Illinois) and Stata 15.1 (Stata Corp LLC, Texas, USA). A two-sided *P* value <0.05 was considered statistically significant.

## Results

### Baseline Characteristics According to Quartiles of TyG Index

The analysis included 10,196 participants at baseline, 61.48% men with a mean age of 62.77 ± 6.63 years. The mean TyG index was 9.49 ± 0.73, and we categorized the included population into four groups based on the quartiles of the baseline TyG index (**Table 1**). Participants with a higher TyG index were younger and more often male and white. They had higher levels of BMI, diastolic blood pressure, total cholesterol (TC), LDL-C, TG, fasting glucose, glycated hemoglobin, eGFR, and serum creatinine, lower levels of education, and were less frequent current drinkers (all *P*<0.001). Likewise, participants in the higher TyG index quartile had a higher prevalence of CVD and CHF. They were more prone to taking metformin drugs and less prone to taking insulin, ACEI/ARB, statin, and cholesterol absorption inhibitors (all *P*<0.001). These findings suggested that a higher TyG index in participants may be associated with more traditional risk factors.

**Table 1:** Baseline characteristics of participants by quartiles of TyG index.

	Total	Q1	Q2	Q3	Q4	*P* value
	(n=10,196)	(n=2,550)	(n=2,550)	(n=2,547)	(n=2,549)	
**Age, years (mean ± SD)**	62.77 ± 6.63	63.512 ± 6.91	63.24 ± 6.61	62.72 ± 6.58	61.57 ± 6.24	<0.001
**TyG index**	9.49 ± 0.73	8.58 ± 0.35	9.25 ± 0.13	9.70 ± 0.14	10.42 ± 0.43	<0.001
**Sex n (%)**						<0.001
** Female**	3,928 (38.52%)	1,000 (39.22%)	990 (38.82%)	988 (38.79%)	950 (37.27%)	
** Male**	6,268 (61.48%)	1,550 (60.78%)	1,560 (61.18%)	1,559 (61.21%)	1,599 (62.73%)	
**Race/ethnicity, n (%)**						<0.001
** White**	6,371 (62.49%)	1,162 (45.57%)	1,530 (60.00%)	1,775 (69.69%)	1,904 (74.70)	
** Non-White**	3,825 (60.04)	1,388 (53.43%)	1,020 (40.00%)	772 (30.31%)	645 (25.30%)	
**Education, n (%)**						
** Less than high school (1)**	1,502 (14.73%)	429 (16.82%)	407 (15.96%)	350 (13.74%)	316 (12.40%)	<0.001
** High school graduate or GED**	2,692 (26.40%)	713 (27.96%)	649 (25.45%)	670 (26.31%)	660 (25.89%)	<0.001
** Some college**	3,343 (32.79%)	761 (29.84%)	812 (31.84%)	868 (34.08%)	902 (35.39%)	<0.001
** College degree or higher**	2,652 (26.01%)	644 (25.25%)	679 (26.63%)	658 (25.83%)	671 (26.32%)	<0.001
**Previous cardiovascular event, n (%)**	3,586 (35.17%)	855 (33.53%)	878 (34.43%)	896 (35.18%)	957 (37.54%)	0.019
**Previous congestive heart failure, n (%)**	489 (4.80%)	105 (4.12%)	107 (4.20%)	127 (4.99%)	150 (5.88%)	0.01
**Duration of diabetes, years (mean ± SD)**	10.80 ± 7.60	12.22 ± 8.24	10.96 ± 7.61	10.26 ± 7.34	9.77 ± 6.91	<0.001
**Live alone, n (%)**	2,065 (20.65%)	533 (20.90%)	499 (19.57%)	531 (20.85%)	502 (19.69%)	0.484
**Depression, n (%)**	2,412 (23.66%)	475 (18.63%)	538 (21.10%)	653 (25.64%)	746 (29.27%)	<0.001
**Cigarette-smoking status, n (%)**						<0.001
** Yes**	5,925 (58.11%)	1401 (54.94%)	1467 (57.53%)	1513 (59.40%)	1544 (60.57%)	
** No**	4,271 (41.89%)	1149 (45.06%)	1083 (42.47%)	1034 (40.60%)	1005 (39.43%)	
**Alcohol status, n (%)**						<0.001
** Yes**	2,434 (23.87%)	556 (21.80%)	636 (24.94%)	626 (24.58%)	616 (24.17%)	
** No**	7,757 (76.08%)	1993 (78.16%)	1912 (74.98%)	1920 (75.38%)	1930 (75.72%)	
**Weight, kg (mean ± SD)**	93.51 ± 18.41	89.94 ± 18.42	93.26 ± 18.11	94.69 ± 18.36	96.29 ± 18.13	<0.001
**Waist circumference, cm (mean ± SD)**	106.73 ± 13.64	103.63± 13.96	106.59 ± 13.48	107.79 ± 13.35	108.94 ± 13.17	<0.001
**Body mass index, kg/m^2^ (mean ± SD)**	32.22 ± 5.42	31.15 ± 5.56	32.18 ± 5.41	32.60 ± 5.31	32.96 ± 5.22	<0.001
**Blood pressure, mmHg (mean ± SD)**						
**Systolic**	136.36 ± 17.11	136.79 ± 17.22	136.16 ± 16.83	135.52 ± 17.37	136.92 ± 16.99	0.0125
**Diastolic**	74.88 ± 10.66	73.63 ± 10.61	74.40 ± 10.47	75.10 ± 10.57	76.40 ± 10.81	<0.001
**Medications, n (%)**						
** Insulin**	3,565 (34.96%)	1,121 (43.96%)	935 (36.67%)	760 (29.84%)	749 (29.38%)	<0.001
** Metformin**	6,519 (63.94%)	1,550 (60.78%)	1,648 (64.63%)	1,695 (66.55%)	1,626 (66.79%)	<0.001
** ACEI/ARB**	7,066 (69.30%)	1,804 (70.75%)	1,810 (70.98%)	1,757 (68.98%)	1,695 (66.50%)	0.001
** Aspirin**	5,552 (54.45%)	1,412 (55.37%)	1,421 (55.73%)	1,384 (54.34%)	1,335 (52.37%)	0.072
** Statin**	6,468 (63.44%)	1,732 (67.92%)	1,681 (65.92%)	1,618 (63.53%)	1,437 (56.38%)	<0.001
**Cholesterol absorption inhibitors**	207 (2.03%)	46 (1.80%)	53 (2.08%)	63 (2.47%)	45 (1.77%)	0.251
**Glycated hemoglobin, % (mean ± SD)**	8.30 ± 1.06	8.04 ± 0.96	8.15 ± 0.96	8.34 ± 1.03	8.67 ± 1.15	<0.001
**Fasting plasma glucose, mg/dL (mean ± SD)**	175.19 ± 56.17	133.00 ± 38.86	164.79± 42.02	183.60 ± 44.72	219.43± 58.59	<0.001
**Potassium, mg/dL (mean ± SD)**	4.47 ± 0.47	4.43 ± 0.46	4.47 ± 0.43	4.50 ± 0.54	4.50 ± 0.44	<0.001
**Serum creatinine, mg/dL (mean ± SD)**	0.91 ± 0.23	0.93 ± 0.23	0.92 ± 0.23	0.91 ± 0.23	0.89 ± 0.24	<0.001
**eGFR, (mL/min/1.73m^2^) (mean ± SD)**	91.05 ± 27.15	91.11 ± 24.21	90.58 ± 25.44	89.54 ± 24.66	92.93 ± 33.19	<0.001
**Plasma triglycerides, mg/dL (mean ± SD)**	190.11 ± 148.40	88.57 ± 29.18	134.60± 36.89	188.78 ± 49.37	348.62 ± 211.93	<0.001
**Total plasma cholesterol, mg/dL (mean ± SD)**	183.29 ± 41.89	166.51 ± 34.40	175.85 ± 35.50	184.97± 37.50	205.91± 205.9	<0.001
**Plasma LDL-C, mg/dL (mean ± SD)**	104.89 ± 33.93	100.67 ± 29.79	105.63± 31.92	107.25 ± 34.12	106.04 ± 38.80	<0.001
**Incident MACEs, n (%)**	1815 (17.80%)	389 (15.25%)	429 (16.82%)	460 (18.06%%)	537 (21.07%)	<0.001

Quartile ranges are Q1 (6.87 to 9.00), Q2 (9.01 to 9.47), Q3 (9.47 to 9.95), and Q4 (9.95 to 13.36).

P value for test of difference across quartiles of triglyceride-glucose (TyG) index using chi-square test (categorical variables) or analysis of variance (continuous variables) or Kruskal-Wallis test (nonparametric comparisons).

eGFR, estimated glomerular filtration rate; LDL-C, low-density lipoprotein cholesterol; ACEI, angiotensin-converting enzyme inhibitor; ARB, angiotensin receptor blocker; MACEs, major adverse cardiovascular events.

### Associations Between Baseline TyG Index and Primary and Secondary Adverse CV Outcomes

**Figure 1:**
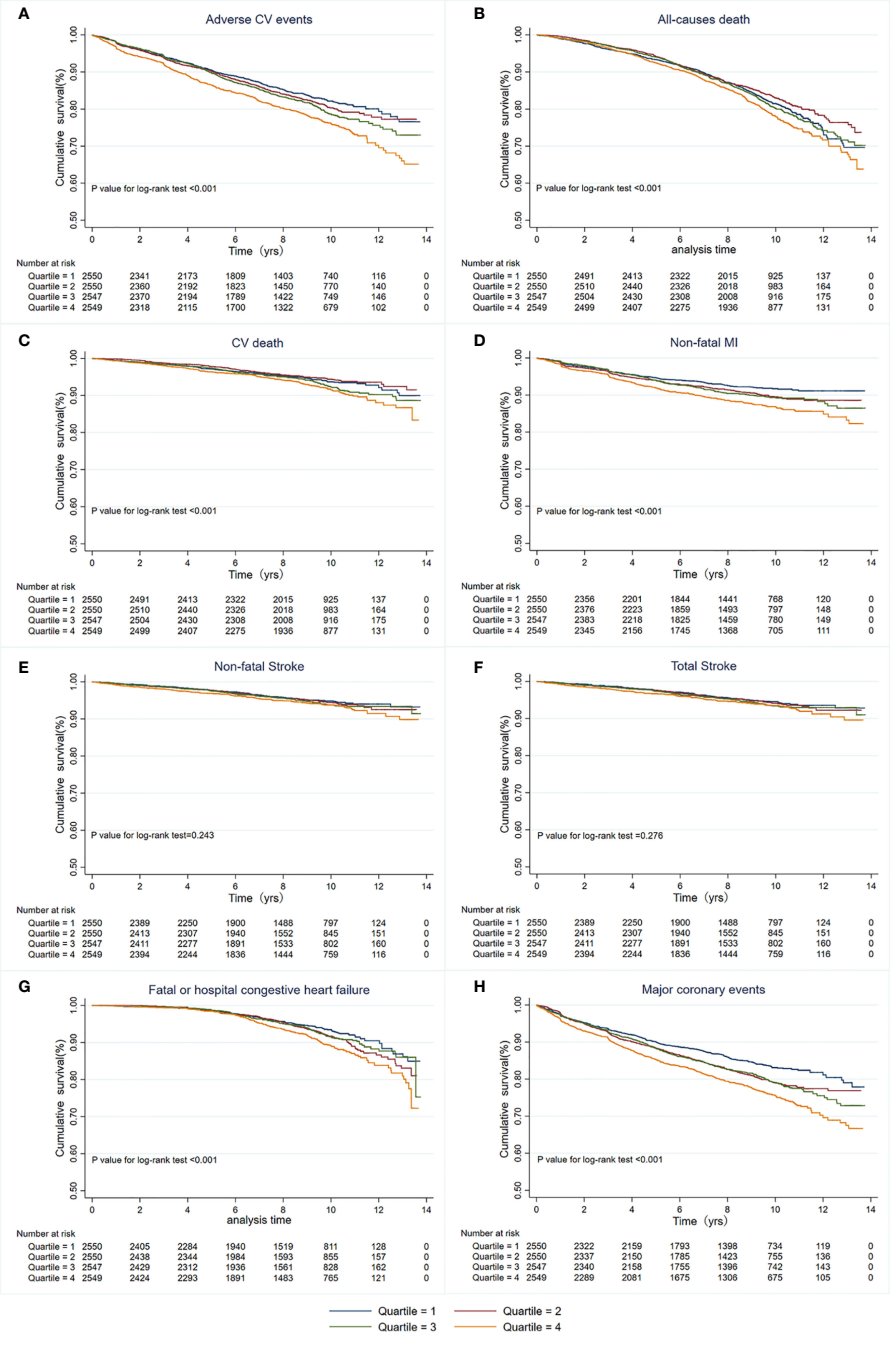
Kaplan-Meier survival curves for primary and secondary outcomes based on quartiles of baseline TyG index. **(A)** MACEs; **(B)** All-cause death; **(C)** CV death; **(D)** Non-fatal MI; **(E)** Non-fatal stroke; **(F)** Total stroke; **(G)** Fatal or hospital congestive heart failure; **(H)** Major coronary events. MACEs, Major adverse cardiovascular events; CV, Cardiovascular; MI, myocardial infarction.

During a median of 8.8 years and a mean of 7.7 years from randomization, 1,815 patients (17.8%) developed primary endpoint events. As **Table 1** shows, the risk of MACEs increased with increasing quartiles of TyG index (quartiles 1-4: 389 [15.25%] vs. 429 [16.82%] vs. 460 [18.06%] vs. 537 [21.07%]; *P*<0.001). In the multivariate model that measured the TyG index as a continuous variable, a 1-SD increase in the TyG index was associated with a 19.00% higher risk of MACEs after full adjustment for potential confounders (HR 1.19, 95% CI 1.11-1.28; *P*<0.001; **Table 2**). The results were similar when we categorized individuals by TyG index quartiles: the highest risk of MACEs was observed in the participants with the highest TyG index quartile, in three different adjusted models (all *P*<0.05, **Table 2**). In the final model, the HR (95% CIs) for MACEs comparing the fourth quartile of TyG index with the first quartile was 1.13 (95% CI, 1.06-1.20) (model 3 in **Table 2** and **Figure 1**). The results were similar when the association between baseline TyG index and all-cause death, CV death, non-fatal MI, non-fatal stroke, total stroke, and fatal or hospital CHF (**Table S2**). Notably, the HRs (95% CIs) in the final model for major coronary events comparing the second, third, and fourth quartiles of the TyG index with the first quartile were 1.22 (95% CI, 1.06-1.41), 1.12 (95% CI, 1.03-1.22), and 1.17 (95% CI, 1.10-1.24), respectively (all *P*<0.05, model 3 in **Table 2** and **Figure 1**). These findings suggest that the baseline TyG index was significantly correlated with major coronary events, including CV death, non-fatal MI, or unstable angina. **Figure S2** shows the restricted cubic splines of the risk of MACEs across TyG index levels. Consistent with the analysis using quartiles of sample distribution, the risk of incident MACEs was increased in participants with a higher TyG index (**Figure S2**).

**Table 2:** Risk of incident MACEs and major coronary events for baseline TyG index.

MACEs (a composite of CV death, non-fatal MI, or non-fatal stroke)									
TyG index	Events/No. at risk	Unadjusted	*P* value	Model 1	*P* value	Model 2	*P* value	Model 3	*P* value
		HR (95% CI)		HR (95% CI)		HR (95% CI)		HR (95% CI)	
Quartile 1	389/2550	Ref		Ref		Ref		Ref	
Quartile 2	429/2550	1.08 (0.94-1.24)	0.250	1.07 (0.93-1.23)	0.318	1.03 (0.90-1.19)	0.645	1.05 (0.91-1.21)	0.505
Quartile 3	460/2547	1.08 (1.01-1.16)	0.023	1.07 (1.00-1.15)	0.041	1.06 (0.98-1.16)	0.134	1.08 (0.99-1.18)	0.055
Quartile 4	537/2549	1.27 (1.08-1.18)	<0.001	1.14 (1.09-1,19)	<0.001	1.12 (1.06-1.20)	<0.001	1.13 (1.06-1.20)	<0.001
Per 1 SD	1815/10196	1.14 (1.09-1.19)	<0.001	1.16 (1.11-1.22)	<0.001	1.17 (1.09-1.26)	<0.001	1.19 (1.11-1.28)	<0.001
**Major coronary events (CV death, non-fatal MI, or unstable angina)**									
TyG index	Events/No. at risk	Unadjusted	*P* value	Model 1	*P* value	Model 2	*P* value	Model 3	*P* value
		HR (95% CI)		HR (95% CI)		HR (95% CI)		HR (95% CI)	
Quartile 1	368/2550	Ref		Ref		Ref		Ref	
Quartile 2	461/2550	1.24 (1.08-1.43)	0.002	1.22 (1.06-1.40)	0.005	1.21 (1.05-1.40)	0.008	1.22 (1.06-1.41)	0.006
Quartile 3	466/2547	1.12 (1.05-1.20)	0.001	1.11 (1.04-1.19)	0.003	1.11 (1.02-1.20)	0.015	1.12 (1.03-1.22)	0.007
Quartile 4	552/2549	1.16 (1.11-1.21)	<0.001	1.16 (1.11-1.22)	<0.001	1.16 (1.09-1.23)	<0.001	1.17 (1.10-1.24)	<0.001
Per 1 SD	1847/10196	1.16 (1.11-1.21)	<0.001	1.17 (1.11-1.22)	<0.001	1.20 (1.12-1.29)	<0.001	1.22 (1.14-1.31)	<0.001

Model 1: Adjusted for baseline age, sex, previous cardiovascular event, race, BMI, education, systolic blood pressure, and diastolic blood pressure.

Model 2: Adjusted for model 1 covariates plus baseline eGFR, HbA1c, total plasma cholesterol, plasma LDL-C, live alone, duration of diabetes and depression.

Model 3: Adjusted for model 2 covariates plus treatment with statins, biguanide, aspirin, ACEI/ARB, and insulin.

BMI, body mass index; LDL-C, low-density lipoprotein cholesterol; ACEI, angiotensin-converting enzyme inhibitor; ARB, angiotensin receptor blocker; CI, confidence interval; HR, hazard ratio; CV, cardiovascular; MACEs, major adverse cardiovascular events; MI, myocardial infarction; TyG, triglyceride-glucose; HbA1c, hemoglobin A1c.

### Associations Between TyG Index Trajectories and Adverse CV or Major Coronary Events

**Figure 2:**
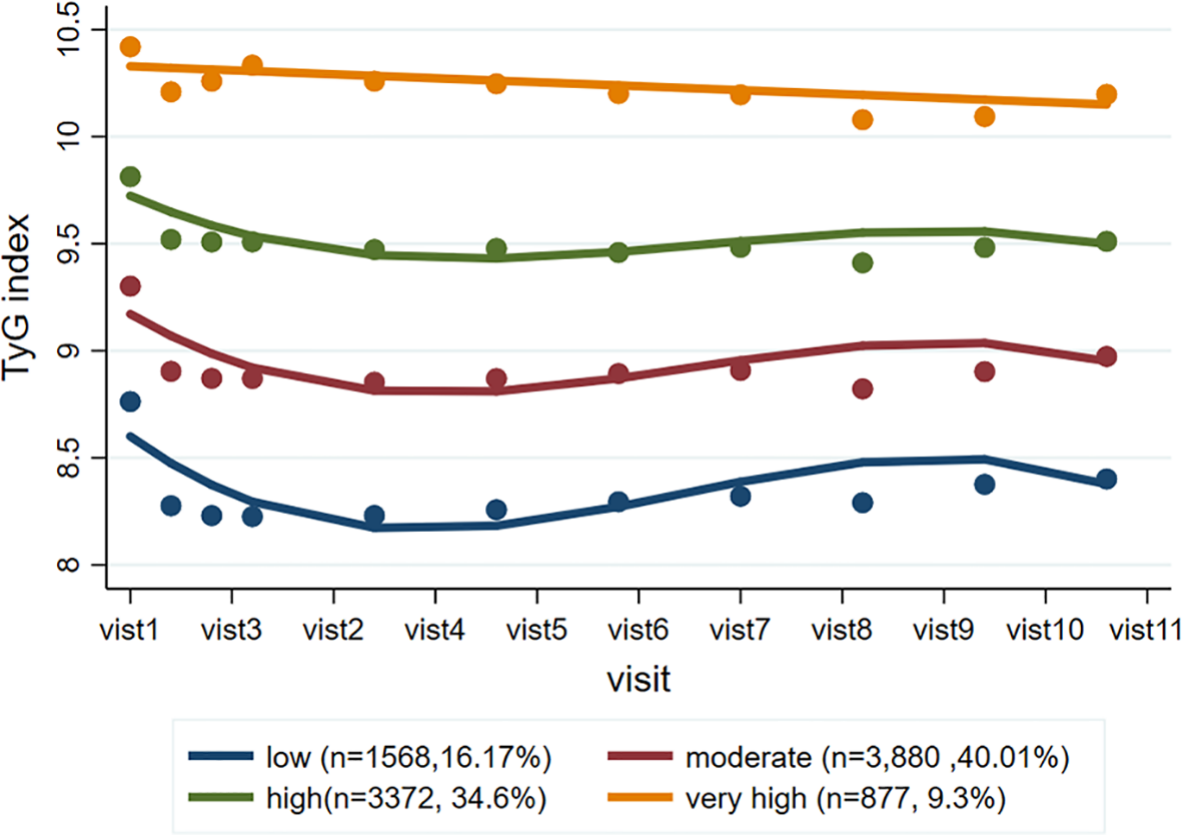
TyG index trajectory groups and percentage of the population in the group. Four discrete trajectories with stable TyG indexes at various levels from visit 1 to visit 11: low (n = 1,568, 16.17%), moderate (n = 3,880, 40.01%), high (n = 3,372, 34.60%), and very high (n = 877, 9.30%) TyG index trajectory groups. TyG, triglyceride-glucose.

A total of 9,697 participants were included in the trajectory analysis (**Figure S1**). Four discrete trajectories with stable TyG indexes at various levels from visit 1 to visit 11 were identified (**Figure 2**): low (n=1,568, 16.17%), moderate (n=3,880, 40.01%), high (n=3,372, 34.60%), and very high (n=877, 9.30%) TyG index trajectory groups. The median (interquartile range) changes in TyG index level during the visits for these trajectory groups were 0.009 (-0.008-0.02) in the low trajectory group, 0.007 (-0.02-0.02) in the moderate trajectory group, -0.007 (-0.03-[-0.003]) in the high trajectory group, 0.007(-0.05 - 0.05) in the very high trajectory group (**Tables S3**). As shown in **Figure S3**, the rates of MACEs were 14.16, 16.57, 19.48, and 22.81% for low, moderate, high, and very high, respectively. TyG index trajectory groups, respectively (*P*<0.001). In addition, the rates of major coronary events were 12.88, 17.22, 19.63, and 25.09% in the low, moderate, high, and very high TyG index trajectory groups, respectively (*P*<0.001). Multivariate Cox regression analyses identified those with TyG index trajectory at high and very high levels as having an even greater risk of MACEs in three different adjusted models (all *P*<0.05, **Table 3**). In the fully adjusted model, compared with those with a low trajectory at a low level, the HRs (95% CIs) for associations of those participants with TyG index trajectories at the moderate, high, and very high levels with the risk of MACEs were 1.20 (95% CI, 1.03-1.41; *P*=0.021), 1.25 (95% CI, 1.15-1.37; *P*<0.001), and 1.26 (95% CI, 1.16-1.37; *P*<0.001), respectively (model 3 in **Table 3**). The results were similar when evaluating the risk of major coronary events for various levels of TyG index trajectory groups. Thus, long-term trajectories of the TyG index identify individuals at a higher risk of MACEs and coronary events in patients with T2DM, indicating that such a population would benefit from more frequent screening for adverse CV events and aggressive risk factor management.

**Table 3:** Risk of adverse CV and major coronary events for various levels of TyG index trajectory groups.

MACEs (a composite of CV death, non-fatal MI, or non-fatal stroke)								
TyG index trajectories	Unadjusted	*P* value	Model 1	*P* value	Model 2	*P* value	Model 3	*P* value
	HR (95% CI)		HR (95% CI)		HR (95% CI)		HR (95% CI)	
Low	Reference		Reference		Reference		Reference	
Moderate	1.21 (1.04-1.41)	0.015	1.21 (1.03-1.40)	0.018	1.20 (1.03-1.41)	0.022	1.20 (1.03-1.41)	0.021
High	1.22 (1.13-1.32)	<0.001	1.23 (1.14-1.33)	<0.001	1.25 (1.15-1.36)	<0.001	1.25 (1.15-1.37)	<0.001
Very high	1.22 (1.14-1.30)	<0.001	1.24 (1.16-1.33)	<0.001	1.25 (1.15-1.35)	<0.001	1.26 (1.16-1.37)	<0.001
**Major coronary events (CV death, non-fatal MI, or unstable angina)**								
TyG index trajectories	Unadjusted	*P* value	Model 1	*P* value	Model 2	*P* value	Model 3	*P* value
	HR (95% CI)		HR (95% CI)		HR (95% CI)		HR (95% CI)	
Low	Reference		Reference		Reference		Reference	
Moderate	1.40 (1.19-1.63)	<0.001	1.37 (1.17-1.60)	<0.001	1.39 (1.18-1.63)	<0.001	1.38 (1.18-1.63)	<0.001
High	1.28 (1.18-1.39)	<0.001	1.29 (1.19-1.40)	<0.001	1.31 (1.20-1.43)	<0.001	1.32 (1.21-1.44)	<0.001
Very high	1.30 (1.22-1.38)	<0.001	1.31 (1.22-1.41)	<0.001	1.33 (1.23-1.45)	<0.001	1.34 (1.24-1.46)	<0.001

Model 1: Adjusted for baseline age, sex, previous cardiovascular event, race, BMI, education, systolic blood pressure, and diastolic blood pressure.

Model 2: Adjusted for model 1 covariates plus baseline eGFR, HbA1c, total plasma cholesterol, plasma LDL-C, live alone, duration of diabetes and depression.

Model 3: Adjusted for model 2 covariates plus treatment with statins, biguanide, aspirin, ACEI/ARB, and insulin.

BMI, body mass index; LDL-C, low-density lipoprotein cholesterol; ACEI, angiotensin-converting enzyme inhibitor; ARB, angiotensin receptor blocker; CI, confidence interval; HR, hazard ratio; CV, cardiovascular; MACEs, major adverse cardiovascular events; MI, myocardial infarction; TyG, triglyceride-glucose; HbA1c, hemoglobin A1c.

## Discussion

This study assessed the association between the baseline TyG index and CV outcomes, which revealed that higher levels of TyG index were significantly associated with an increased risk of MACEs in patients with T2DM during ~10 years of follow-up. More precisely, the TyG index was strongly associated with major coronary events, suggesting that TyG was closely related to the formation and development of atherosclerosis and finally showed a significant increase in the incidence of clinical coronary events with a high TyG index. Furthermore, we identified that the four distinct trajectories of the TyG index confer different risks of MACEs, and a decade trajectory with an elevated TyG index carries a greater risk of future incident MACEs. These findings suggest a potential role for a long-lasting high level of TyG in the pathogenesis of CVD. Therefore, frequent screening for adverse cardiovascular events and aggressive risk factor management in these patients would be highly beneficial.

IR, a key feature of metabolic syndrome and T2DM, is considered a significant risk factor for CVD (23, 24). It has been well-established that IR and coexisting hyperinsulinemia are implicated in the development of dyslipidemia, hypertension, hypercoagulability, and atherosclerosis (25, 26). These metabolic changes caused by IR may promote the development of CVD. In particular, chronic hyperglycemia induced by IR causes oxidative stress. Subsequently, it triggers an inflammatory response that promotes vascular cell damage (26–28). IR leads to elevated plasma triglycerides, reduced plasma HDL-C, and the appearance of small dense LDL-C particles (29). In addition, IR has been implicated in decreased fibrinolytic activity and increased thrombotic events (30, 31). It is worth noting that IR could promote atherosclerosis not only through mechanisms that involve systemic factors, such as dyslipidemia, hypertension, and a proinflammatory state, but also through the effect of perturbed insulin signaling at the level of the intimal cells (26, 32). Thus, there is a known concept on the “common soil” between diabetes mellitus and cardiovascular disease referring to both conditions share common genetic and environmental antecedents (33, 34).These studies have indicated the importance of IR in atherogenesis and advanced plaque progression (26, 35, 36). However, insulin concentrations are not routinely measured in clinical settings and are similar in the ACCORD trial. Therefore, a number of surrogate markers of IR have been proposed and compared with the gold standard of the hyperinsulinemic-euglycemic clamp (37). The TyG index, which is calculated using fasting TG and fasting glucose levels, is a reliable measure of IR (17, 22). The TyG index has been proven to be highly correlated with the euglycemic-hyperinsulinemic clamp formation (22), and thus has a validity similar to HOMA-IR (38). Thus, the TyG index was used as a biomarker of IR in this *post hoc* analysis to study its relationship with the risk of MACEs in patients with diabetes. Moreover, the TyG index has the advantage of being easily accessible in any clinical setting and making our findings usable.

Our study showed that traditional risk factors such as higher BMI, hypertension, and hyperlipidemia were more obvious among participants in higher quartiles of the TyG index. Recently, a mediation analysis was performed to quantify the magnitude and relative contributions of several traditional or non-traditional CV risk factors in the pathway from T2DM to increased CV events, and demonstrated that the most important pathway contributing to CV events was the presence of IR assessed by the TyG index, followed by elevated TG, the presence of microalbuminuria, and reduced kidney function, but not mediated by elevated systolic blood pressure or high LDL-C (39). These data suggest that TyG may further explain the increased CV and mortality risk caused by T2DM. In addition, clinical studies were conducted to investigate the association between TyG index and CVD morbidity and mortality. A cross-sectional study, including 888 asymptomatic T2DM patients without CHD, showed that a higher TyG index was associated with an increased risk of significant coronary artery stenosis (40). Moreover, a nested case-control study of 1,282 T2DM patients with stable coronary artery disease showed that the TyG index was positively associated with future CV events, defined as all-cause death, non-fatal MI, stroke, and post-discharge revascularization (41). In line with previous studies, our study showed that individuals with the highest quartile of the baseline TyG index had a higher risk of MACEs than those with the lowest quartile. Meanwhile, the association remained statistically significant after adjusting for all the aforementioned risk factors. These findings suggest that the clinical management of the TyG index may have additional effects on CVD development, even under vigorous control of traditional risk factors.

Given that TyG index levels may vary over time, previous studies based on the TyG index measured at a single time point may not reflect long-term exposure. Thus, longitudinal measurements and recording of TyG indices to identify TyG index trajectories are required. It is feasible that the participant’s electronic medical record allows the rapid integration of data across multiple time points. Because the trajectories of TyG represent an added value to the baseline levels to plan and monitor participant follow-up, a recent study evaluated the influence of baseline TyG index and different trajectory changes over 20 years on the development of peripheral artery disease and found that a two-decade trajectory with an elevated TyG index carried a greater risk of future peripheral artery disease incidence (42). Therefore, measurements of the long-term trajectories of the TyG index provide reliable and robust results. Our study assessed the impact of long-lasting IR at various levels for the first time using the TyG index for future MACEs in patients with T2DM. Our findings revealed heterogeneous patterns of trends in the TyG index within the ACCORD population. The baseline TyG index levels cannot fully depict this dynamic change in the trend over time. In addition, the risk for individuals may change during follow-up. Thus, the TyG index trajectory reflects the long-term impact of the TyG index on adverse CV outcomes. In addition, our study suggests that those trajectory groups with long-term high and very high TyG index levels are at a greater risk of MACEs over 10 years after adjustment for traditional risk factors. We can graph trends in the TyG index to identify high-risk individuals who behave similarly to those with TyG index trajectories at high and very high levels observed in the present analysis. Such a population may benefit from earlier and more frequent screening for adverse CV events and aggressive risk factor management, such as control of blood pressure, cessation of smoking, and maintenance of metabolic health.

Nonetheless, this study also has some limitations: First, we could not exclude the possibility of residual confounders despite our careful adjustment for the well-known and suspected risk factors due to the nature of any observational studies. Second, patients included in the study were mainly Caucasians aged 40-79 years at baseline. Thus, it may differ outside this age range and in other ethnicities. Finally, we could not compare the trajectories of the TyG index with HOMA-IR for predicting adverse CV events due to missing records of insulin levels in the ACCORD/ACCORDION study. Despite these limitations that may interfere with the clinical application of the TyG threshold found in our study, we show it is necessary to strictly monitor lipid and glucose levels in T2DM patients during long-term follow-up.

In conclusion, the TyG index was significantly and positively associated with adverse CV outcomes, which suggested that the TyG index may be a valuable predictor of MACEs in patients with T2DM. More importantly, long-term trajectories of the TyG index could identify individuals at a higher risk of MACEs among patients with T2DM who require specific preventive and therapeutic approaches.

## Data Availability Statement

The original contributions presented in the study are included in the article/**Supplementary Material**. Further inquiries can be directed to the corresponding author.

## Ethics Statement

The studies involving human participants were reviewed and approved by the Wake Forest University, USA (coordinating center) and the institutional review boards at each center (participating clinical sites). The patients/participants provided their written informed consent to participate in this study.

## Author Contributions

This study was completed in collaboration with the following authors: LF and ST designed the study theme and methods. LF, NZ, and YZ collected and analyzed the data. ST wrote the paper. ZX, ST, and YW edited the paper. All authors read and approved the final manuscript.

## Funding

This research was supported by the National Natural Science Foundation of China (81801394 to ST) and the Natural Science Foundation of Hunan Province (2019JJ50878 to ST).

## Conflict of Interest

The authors declare that the research was conducted in the absence of any commercial or financial relationships that could be construed as a potential conflict of interest.

## Publisher’s Note

All claims expressed in this article are solely those of the authors and do not necessarily represent those of their affiliated organizations, or those of the publisher, the editors and the reviewers. Any product that may be evaluated in this article, or claim that may be made by its manufacturer, is not guaranteed or endorsed by the publisher.

## Supplementary Material

Supplementary Figure 1 Flowchart for selection of study participants from the Action to Control Cardiovascular Risk in Diabetes. Enrollment chart demonstrating patients included in the final sample; ACCORD, Action to Control Cardiovascular Risk in Diabetes; TyG, triglyceride-glucose; CV, cardiovascular.

Supplementary Figure 2 Adjusted hazard ratios of primary outcome according to baseline TyG index. **(A)** Histograms represent the frequency distribution of baseline TyG index. **(B)** The hazard ratio is adjusted for baseline age, sex, previous cardiovascular event, race, BMI, education, systolic blood pressure, diastolic blood pressure, eGFR, HbA1c, total plasma cholesterol, plasma LDL-C, live alone, duration of diabetes, depression, statin, biguanide, aspirin, ACEI/ARB, and insulin. Red solid line represents the hazard ratio of TyG index across the whole range. Gray dotted lines represent the 95% confidence interval. BMI, body mass index; eGFR, estimated glomerular filtration rate; HbA1c, hemoglobin A1c; LDL-C, low-density lipoprotein cholesterol; TyG, triglyceride-glucose; ACEI/ARB, angiotensin converting enzyme inhibitor/angiotensin-receptor blocker.

Supplementary Figure 3 Prevalence of cardiovascular events across the TyG index trajectory groups. **(A)** Prevalence of MACEs across the TyG index trajectory groups; **(B)** Prevalence of major coronary events across the TyG index trajectory groups. TyG: triglyceride-glucose; MACEs: Major adverse cardiovascular events

Supplementary Table 1 Group-based trajectory model fit summary (N=9,697).

Supplementary Table 2 Risk of incident secondary outcomes for baseline TyG index. Model 1: Adjusted for baseline age, sex, previous cardiovascular event, race, BMI, education, systolic blood pressure, and diastolic blood pressure. Model 2: Adjusted for model 1 covariates plus baseline eGFR, HbA1c, total plasma cholesterol, plasma LDL-C, live alone, duration of diabetes and depression. Model 3: Adjusted for model 2 covariates plus treatment with statins, biguanide, aspirin, ACEI/ARB, and insulin. BMI, body mass index; LDL-C, low-density lipoprotein cholesterol; ACEI, angiotensin-converting enzyme inhibitor; ARB, angiotensin receptor blocker; CI, confidence interval; HR, hazard ratio; CV, cardiovascular; MI, myocardial infarction; TyG, triglyceride-glucose; HbA1c, hemoglobin A1c.

Supplementary Table 3 TyG index at examination visits by trajectory groups of TyG index. TyG, triglyceride-glucose.
