# Supplementary figures and images for: Impact of Baseline and Trajectory of Triglyceride-Glucose Index on Cardiovascular Outcomes in Patients With Type 2 Diabetes Mellitus
Source: Front Endocrinol (Lausanne). 2022 Mar 24;13:858209. doi: 10.3389/fendo.2022.858209 (PMC8987353; doi:10.3389/fendo.2022.858209)

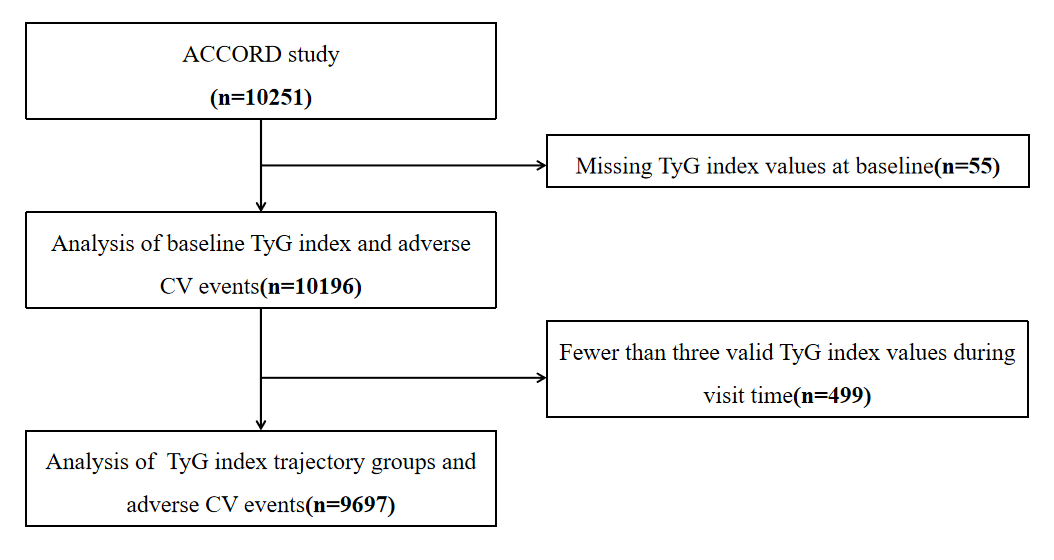

Supplement: Supplementary Figure 1 — Flowchart for selection of study participants from the Action to Control Cardiovascular Risk in Diabetes. Enrollment chart demonstrating patients included in the final sample; ACCORD, Action to Control Cardiovascular Risk in Diabetes; TyG, triglyceride-glucose; CV, cardiovascular. [file Image_1.tif]

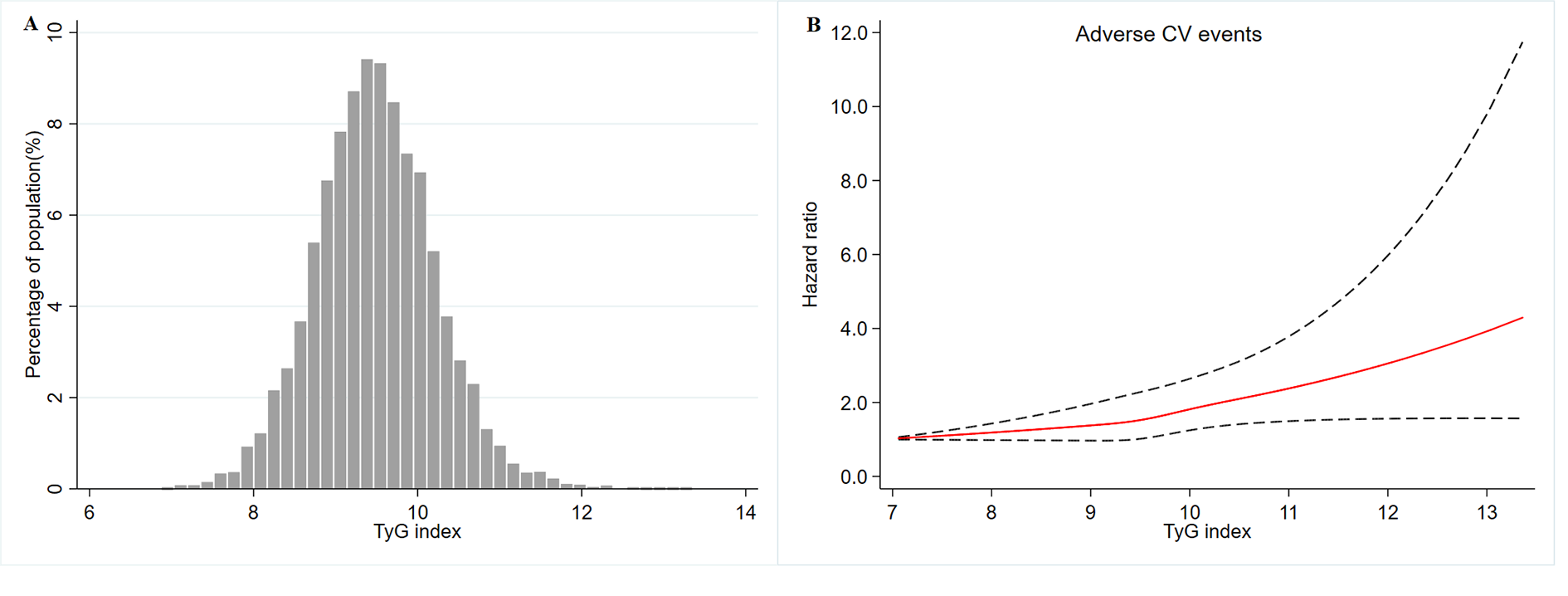

Supplement: Supplementary Figure 2 — Adjusted hazard ratios of primary outcome according to baseline TyG index. (A) Histograms represent the frequency distribution of baseline TyG index. (B) The hazard ratio is adjusted for baseline age, sex, previous cardiovascular event, race, BMI, education, systolic blood pressure, diastolic blood pressure, eGFR, HbA1c, total plasma cholesterol, plasma LDL-C, live alone, duration of diabetes, depression, statin, biguanide, aspirin, ACEI/ARB, and insulin. Red solid line represents the hazard ratio of TyG index across the whole range. Gray dotted lines represent the 95% confidence interval. BMI, body mass index; eGFR, estimated glomerular filtration rate; HbA1c, hemoglobin A1c; LDL-C, low-density lipoprotein cholesterol; TyG, triglyceride-glucose; ACEI/ARB, angiotensin converting enzyme inhibitor/angiotensin-receptor blocker. [file Image_2.tif]

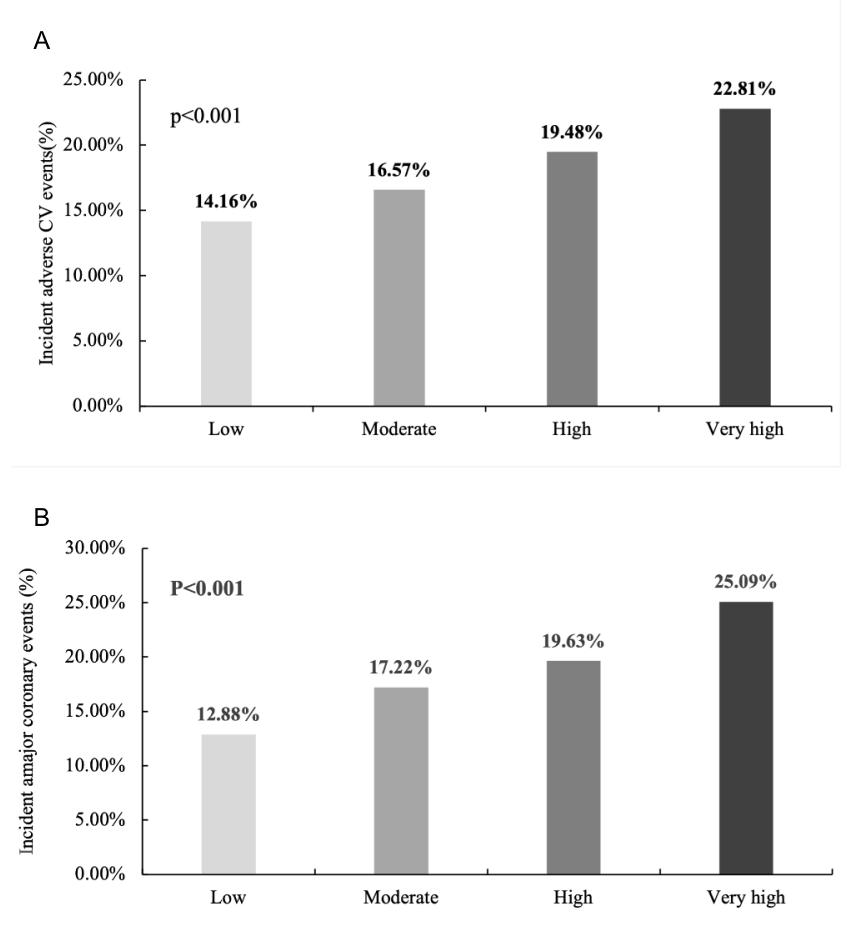

Supplement: Supplementary Figure 3 — Prevalence of cardiovascular events across the TyG index trajectory groups. (A) Prevalence of MACEs across the TyG index trajectory groups; (B) Prevalence of major coronary events across the TyG index trajectory groups. TyG: triglyceride-glucose; MACEs: Major adverse cardiovascular events [file Image_3.tif]
