# Supplementary material for: Impact of Baseline and Trajectory of Triglyceride-Glucose Index on Cardiovascular Outcomes in Patients With Type 2 Diabetes Mellitus
Source: Front Endocrinol (Lausanne). 2022 Mar 24;13:858209. doi: 10.3389/fendo.2022.858209 (PMC8987353; doi:10.3389/fendo.2022.858209)
Supplement: Supplementary Table 1 — Group-based trajectory model fit summary (N=9,697). [file Table_1.docx]

**Table S1. Group-based trajectory model fit summary (N=9,697)**

| Model | AIC | BIC (n=68273) | BIC (n=9697) | Average posterior probability |
| --- | --- | --- | --- | --- |
| 2 | 65079.10 | 65124.76 | 65115.00 | 0.95/0.95 |
| 3 | 61377 | 61445 | 61431.22 | 0.93/0.91/0.93 |
| 4 | 59957.90 | 60040.08 | 60022.52 | 0.89/0.87/0.89/0.92 |
| 5 | 59491.89 | 59583.21 | 59563.69 | 0.88/0.86/0.85/0.84/0.89 |

**Table S2. Risk of incident secondary outcomes for baseline TyG index**

**All-causes mortality**

| TyG index | Events/No. at risk | Unadjusted  HR (95% CI) | *P* value | Model 1  HR (95% CI) | *P* value | Model 2  HR (95% CI) | *P* value | Model 3  HR (95% CI) | *P* value |
| --- | --- | --- | --- | --- | --- | --- | --- | --- | --- |
| Quartile 1 | 472/2550 | Ref |  | Ref |  | Ref |  | Ref |  |
| Quartile 2 | 435/2550 | 0.90 (0.79-1.03) | 0.124 | 0.90 (0.79-1.03) | 0.120 | 0.90 (0.78-1.03) | 0.126 | 0.92 (0.80-1.054) | 0.233 |
| Quartile 3 | 492/2547 | 1.02 (0.96-1.08) | 0.577 | 1.03 (0.96-1.10) | 0.411 | 1.03 (0.95-1.11) | 0.493 | 1.04 (0.96-1.13) | 0.291 |
| Quartile 4 | 547/2549 | 1.06 (1.02-1.10) | 0.007 | 1.09 (1.05-1.14) | <0.001 | 1.07 (1.02-1.14) | 0.013 | 1.09 (1.03-1.15) | 0.004 |
| Per 1 SD | 1946/10196 | 1.07 (1.02-1.12) | 0.003 | 1.12 (1.07-1.17) | <0.001 | 1.09 (1.02-1.16) | 0.014 | 1.10 (1.03-1.18) | 0.004 |

**CV death**

| TyG index | Events/No. at risk | Unadjusted  HR (95% CI) | *P* value | Model 1  HR (95% CI) | *P* value | Model 2  HR (95% CI) | *P* value | Model 3  HR (95% CI) | *P* value |
| --- | --- | --- | --- | --- | --- | --- | --- | --- | --- |
| Quartile 1 | 148/2550 | Ref |  | Ref |  | Ref |  | Ref |  |
| Quartile 2 | 136/2550 | 0.90 (0.71-1.14) | 0.391 | 0.90 (0.71-1.14) | 0.372 | 0.88 (0.69-1.12) | 0.296 | 0.90 (0.70-1.14) | 0.382 |
| Quartile 3 | 178/2547 | 1.09 (0.98-1.22) | 0.109 | 1.09 (0.97-1.22) | 0.139 | 1.10 (0.96-1.25) | 0.164 | 1.13 (0.99-1.29) | 0.072 |
| Quartile 4 | 201/2549 | 1.11 (1.04-1.20) | 0.003 | 1.13 (1.04-1.21) | 0.002 | 1.15 (1.04-1.27) | 0.005 | 1.16 (1.05-1.30) | 0.003 |
| Per 1 SD | 663/10196 | 1.15 (1.07-1.24) | <0.001 | 1.18 (1.09-1.28) | <0.001 | 1.26 (1.11-1.42) | <0.001 | 1.26 (1.12-1.42) | <0.001 |

**Non-fatal MI**

| TyG index | Events/No. at risk | Unadjusted HR (95% CI) | *P* value | Model 1  HR (95% CI) | *P* value | Model 2  HR (95% CI) | *P* value | Model 3  HR (95% CI) | *P* value |
| --- | --- | --- | --- | --- | --- | --- | --- | --- | --- |
| Quartile 1 | 181/2550 | Ref |  | Ref |  | Ref |  | Ref |  |
| Quartile 2 | 228/2550 | 1.24 (1.02-1.51) | 0.030 | 1.22 (1.00-1.49) | 0.045 | 1.20 (0.98-1.47) | 0.086 | 1.22 (0.99-1.49) | 0.061 |
| Quartile 3 | 232/2547 | 1.13 (1.02- 1.24) | 0.015 | 1.12 (1.02-1.24) | 0.019 | 1.07 (0.96-1.21) | 0.226 | 1.10 (0.98-1.24) | 0.112 |
| Quartile 4 | 291/2549 | 1.18 (1.11-1.26) | <0.001 | 1.19 (1.12-1.27) | <0.001 | 1.18 (1.09-1.28) | <0.001 | 1.19 (1.10-1.29) | <0.001 |
| Per 1 SD | 932/10196 | 1.18 (1.11-1.26) | <0.001 | 1.19 (1.12-1.28) | <0.001 | 1.21 (1.10-1.33) | <0.001 | 1.23 (1.12-1.36) | <0.001 |

**Non-fatal Stroke**

| TyG index | Events/No. at risk | Unadjusted  HR (95% CI) | *P* value | Model 1  HR (95% CI) | *P* value | Model 2  HR (95% CI) | *P* value | Model 3  HR (95% CI) | *P* value |
| --- | --- | --- | --- | --- | --- | --- | --- | --- | --- |
| Quartile 1 | 108/2550 | Ref |  | Ref |  | Ref |  | Ref |  |
| Quartile 2 | 118/2550 | 1.06 (0.82-1.38） | 0.653 | 1.05 (0.81-1.37) | 0.711 | 0.97 (0.74-1.28) | 0.842 | 0.99 (0.75-1.29) | 0.915 |
| Quartile 3 | 123/2547 | 1.06 (0.93-1.21） | 0.382 | 1.06 (0.93-1.21) | 0.383 | 1.09 (0.94-1.28) | 0.257 | 1.10 (0.94-1.29) | 0.232 |
| Quartile 4 | 136/2549 | 1.09 (1.00-1.18） | 0.057 | 1.09 (1.00-1.19) | 0.044 | 1.05 (0.94-1.17) | 0.403 | 1.05 (0.94-1.18) | 0.387 |
| Per 1 SD | 485/10196 | 1.11 (1.01-1.21) | 0.024 | 1.12 (1.02-1.23) | 0.014 | 1.10 (0.97-1.26) | 0.146 | 1.12 (0.98-1.28) | 0.102 |

**Total Stroke**

| TyG index | Events/No. at risk | Unadjusted  HR (95% CI) | *P* value | Model 1  HR (95% CI) | *P* value | Model 2  HR (95% CI) | *P* value | Model 3  HR (95% CI) | *P* value |
| --- | --- | --- | --- | --- | --- | --- | --- | --- | --- |
| Quartile 1 | 115/2550 | Ref |  | Ref |  | Ref |  | Ref |  |
| Quartile 2 | 124/2550 | 1.05 (0.81-1.35) | 0.720 | 1.04 (0.81-1.35) | 0.749 | 0.97 (0.75-1.27) | 0.838 | 0.98 (0.75-1.28) | 0.897 |
| Quartile 3 | 132/2547 | 1.06 (0.94-1.21) | 0.334 | 1.06 (0.93-1.20) | 0.380 | 1.10 (0.95-1.28) | 0.218 | 1.11 (0.95-1.29) | 0.185 |
| Quartile 4 | 142/2549 | 1.08 (0.99-1.17) | 0.072 | 1.09 (1.00-1.18) | 0.057 | 1.05 (0.94-1.17) | 0.409 | 1.05 (0.94-1.17) | 0.420 |
| Per 1 SD | 513/10196 | 1.10 (1.01-1.20) | 0.030 | 1.12 (1.02-1.22) | 0.014 | 1.11 (0.97-1.26) | 0.118 | 1.12 (0.98-1.28) | 0.084 |

**Fatal or hospital congestive heart failure**

| TyG index | Events/No. at risk | Unadjusted  HR (95% CI) | *P* value | Model 1  HR (95% CI) | *P* value | Model 2  HR (95% CI) | *P* value | Model 3  HR (95% CI) | *P* value |
| --- | --- | --- | --- | --- | --- | --- | --- | --- | --- |
| Quartile 1 | 139/2550 | Ref |  | Ref |  | Ref |  | Ref |  |
| Quartile 2 | 177/2550 | 1.21 (0.97-1.52) | 0.087 | 1.14 (0.91-1.43) | 0.242 | 1.13 (0.90-1.42) | 0.305 | 1.15 (0.91-1.45) | 0.241 |
| Quartile 3 | 163/2547 | 1.07 (0.95-1.20) | 0.257 | 1.05 (0.94-1.18) | 0.371 | 1.03 (0.90-1.18) | 0.679 | 1.05 (0.91-1.20) | 0.515 |
| Quartile 4 | 212/2549 | 1.16 (1.08-1.25) | <0.001 | 1.19 (1.10-1.28) | <0.001 | 1.16 (1.06-1.28) | 0.002 | 1.17 (1.07-1.29) | 0.001 |
| Per 1 SD | 691/10196 | 1.18 (1.10-1.27) | <0.001 | 1.22 (1.12-1.32) | <0.001 | 1.23 (1.10-1.38) | <0.001 | 1.25 (1.11-1.40) | <0.001 |

Model 1: Adjusted for baseline age, sex, previous cardiovascular event, race, BMI, education, systolic blood pressure, and diastolic blood pressure

Model 2: Adjusted for model 1 covariates plus baseline eGFR, HbA1c, total plasma cholesterol, plasma LDL-C, live alone, duration of diabetes and depression

Model 3: Adjusted for model 2 covariates plus treatment with statins, biguanide, aspirin, ACEI/ARB, and insulin.

BMI, body mass index; LDL-C, low-density lipoprotein cholesterol; ACEI, angiotensin-converting enzyme inhibitor; ARB, angiotensin receptor blocker; CI, confidence interval; HR, hazard ratio; CV, cardiovascular; MI, myocardial infarction; TyG, triglyceride-glucose; HbA1c, hemoglobin A1c.

**Table S3. TyG index at examination visits by trajectory groups of TyG index**

|  | TyG index trajectory groups | | | |
| --- | --- | --- | --- | --- |
|  | Low | Moderate | High | Very high |
| Visit 1 | 8.74±0.55 | 9.29±0.53 | 9.82±0.54 | 10.45±0.65 |
| Visit 2 | 8.26±0.46 | 8.90±0.47 | 9.53±0.94 | 10.23±0.57 |
| Visit 3 | 8.21±0.45 | 8.87±0.44 | 9.52±0.47 | 10.29±0.58 |
| Visit 4 | 8.20±0.43 | 8.86±0.44 | 9.52±0.49 | 10.36±0.58 |
| Visit 5 | 8.21±0.44 | 8.85±0.45 | 9.49±0.48 | 10.28±0.58 |
| Visit 6 | 8.23±0.44 | 8.87±0.45 | 9.49±0.47 | 10.26±0.59 |
| Visit 7 | 8.28±0.47 | 8.89±0.45 | 9.47±0.48 | 10.22±0.61 |
| Visit 8 | 8.31±0.49 | 8.90±0.46 | 9.49±0.52 | 10.21±0.59 |
| Visit 9 | 8.28±0.44 | 8.82±0.46 | 9.42±0.49 | 10.10±0.59 |
| Visit 10 | 8.37±0.48 | 8.90±0.46 | 9.49±0.55 | 10.12±0.56 |
| Visit 11 | 8.39±0.47 | 8.96±0.47 | 9.53±0.53 | 10.21±0.66 |
| Change from Visit 1 to Visit 11 | 0.009 (-0.008-0.02) | 0.007 (-0.02-0.02) | -0.007 (-0.03- -0.003) | 0.007 (-0.05-0.05) |

TyG, triglyceride-glucose
